# Recognising atypical presentations of benign acute childhood myositis: Insights from a case series and literature review

**DOI:** 10.51866/cr.805

**Published:** 2025-08-09

**Authors:** Syed Ahmed Zaki

**Affiliations:** 1 MBBS, MD, FRCPCH, Associate Professor, Department of Paediatrics, All India Institute of Medical Sciences, Bibinagar, Telangana, India. E-mail: drzakisyed@gmail.com

**Keywords:** Benign acute childhood myositis, Toe walking, Crawling, Recurrence, Rhabdomyolysis

## Abstract

Benign acute childhood myositis (BACM) is a rare, self-limiting inflammatory condition affecting the skeletal muscle, typically emerging during the early convalescent phase of viral infections. Most patients recover with bed rest, analgesics and adequate hydration. However, atypical presentations can sometimes lead to delayed diagnoses and unnecessary investigations. We report five cases of BACM with unusual presentations at admission: toe walking, crawling, delayed recovery, rhabdomyolysis and recurrence. All patients experienced favourable outcomes and complete recovery upon follow-up. This case series highlights the diverse presentations of BACM, emphasising the importance of increasing awareness for early diagnosis, reducing unnecessary workups and providing proper counselling for parents.

## Introduction

Benign acute childhood myositis (BACM) is a rare, transient and self-limiting condition commonly observed in school-age children.^1^ First described by Ake Lundberg in 1957 as ‘myalgia cruris epidemica’, BACM typically arises during the early convalescent phase of various viral infections, presenting with severe calf pain and an inability to walk.^1,2^ Multiple viral agents have been linked to its pathogenesis, including influenza virus, adenovirus, coxsackievirus, dengue virus and parainfluenza.^1,3^ Majority follow a benign course, with complete recovery typically occurring within a week.^1^ However, atypical presentations can sometimes lead to diagnostic challenges.^4-6^ By recognising the wide range of presentations in BACM, from mild to more severe symptoms, physicians can ensure that children receive appropriate care and counselling while avoiding unnecessary interventions.

We report five cases of BACM with unusual presentations upon admission to a tertiary care hospital over a 2-year period.

## Case presentation

### Case 1

A previously healthy 5-year-old boy presented with toe walking and severe calf pain lasting 1 day. He had experienced an upper respiratory tract infection (URTI) for 3 days and intermittent bilateral lower limb pain for 2 days. On the morning of admission, he was unable to get out of bed due to intense calf pain. Upon standing, he ambulated only on his toes and could not walk flat-footed. Minimal weakness in the bilateral hip and knee flexors was noted, with his feet held in a plantar-flexed position and significant heel cord tightening, preventing both active and passive dorsiflexion. Bilateral calf muscle tenderness was present, and his creatine kinase (CK) level was 1536 U/L (normal range: 17-193 U/L). His nasopharyngeal swab test was positive for influenza A virus. He received maintenance intravenous fluids (IVFs) and analgesics. Over the next 2 days, his walking improved gradually; while some toe walking was still observed intermittently, the heel cords became looser. He was discharged on the third day with a normal gait. At follow-up 1 week later, he reported no pain or weakness, and his repeat CK levels were normal.

### Case 2

A previously healthy 12-year-old girl presented with a URTI for 4 days, severe generalised myalgia for 2 days and dark urine for 1 day. Examination revealed tenderness in both arms and legs. She was started on aggressive IVFs (twice maintenance) and analgesics. Laboratory results indicated a serum creatinine level of 1.1 mg/dL, serum myoglobin level of 790 ng/mL (normal range: 28-72 ng/mL) and CK level of 11,836 U/L. A nephrology consultation was requested due to the elevated CK and serum creatinine levels. Her nasopharyngeal swab test was positive for influenza B virus. The patient was advised to continue aggressive fluid administration with normal saline to prevent acute kidney injury (AKI). IVFs were titrated to maintain a urine output of 3-4 mL/kg/h, and the CK levels were monitored serially. Over the next 3 days, the CK level decreased to 4234 U/L. She showed symptomatic improvement and was passing normal urine. She was discharged on the ninth day. At follow-up 2 weeks later, she was asymptomatic with normal CK, urine and serum creatinine levels.

### Case 3

A previously healthy 8-year-old boy presented with a URTI for 4 days and bilateral calf pain for 2 days. He was unable to walk and had been crawling at home for ambulation. He arrived at the hospital in a wheelchair, with a CK level of 2842 U/L. His nasopharyngeal swab test was positive for adenovirus. He received maintenance IVFs and analgesics. Over the next 2 days, his ability to walk improved, and he was discharged on the fourth day with a normal gait. At follow-up 1 week later, he was asymptomatic with a normal gait and CK level.

### Case 4

A previously healthy 4-year-old boy presented with a URTI for 3 days and calf pain for 1 day. His father reported a similar episode of lower limb pain and weakness following a URTI 1 year prior, during which a nasopharyngeal swab was positive for adenovirus. His CK level was 2035 U/L. The previous episode had resolved within 3-4 days with symptomatic treatment. In the current episode, his CK level was 1235 U/L, and a nasopharyngeal swab was positive for influenza A virus. He was advised to rest, take analgesics and stay hydrated on an outpatient basis. At follow-up 1 week later, he was asymptomatic with a normal CK level.

### Case 5

A previously healthy 10-year-old girl presented with a URTI for 5 days and calf pain for 2 days. Examination revealed tenderness in the calf muscles and mild weakness in the lower limbs (4/5), while other findings were normal. Her CK level was 2239 U/L; vitamin D level, 4 ng/ mL; and serum calcium level, 1.5 mmol/L. Her nasopharyngeal swab was positive for influenza B virus. She was started on maintenance IVFs, analgesics and vitamin D and calcium supplements. Although her respiratory symptoms subsided 2 days after admission, calf pain and gait abnormalities persisted. She was followed up weekly on an outpatient basis. Her CK level showed a decreasing trend and returned to normal at the 4-week follow-up. Although recovery was delayed, she eventually achieved complete recovery after 4 weeks.

The clinical and laboratory features of these cases are summarised in Table 1.

**Table 1:** Clinical and laboratory manifestations of the cases.

Tests	Case 1	Case 2	Case 3	Case 4	Case 5
**Preceding URTI**	Yes	Yes	Yes	Yes	Yes
**Bilateral calf pain**	Yes	Yes	Yes	Yes	Yes
**Deep tendon reflex**	Normal	Normal	Normal	Normal	Normal
**Muscle power on admission as per the MRC scale**	4/5 in all four limbs	4/5 in all four limbs	4/5 in all four limbs	5/5 in all four limbs	4/5 in all four limbs
**Inability to walk**	Yes	Yes	Yes	No	No
**Haemoglobin level (g/dL)**	9.2	10.4	9.8	11.2	8.6
**Total leucocyte count (cells/mm^3^)**	3600	18,990	5600	2400	5400
**Platelet count (platelets/mm^3^)**	126 * 10^3^	114 * 10^3^	280 * 10^3^	106 * 10^3^	156 * 10^3^
**SGPT level (N: 7-56 IU/L)**	68	254	72	60	57
**SGOT level (N: 5-40 IU/L)**	91	1345	88	78	70
**Creatine kinase level (N: 17-193 U/L)**	1536	11,836	2842	1235	2239
**Nasopharyngeal swab test result**	Influenza A	Influenza B	Adenovirus	Influenza A	Influenza B
**Treatment interventions**	Intravenous fluids (maintenance) Analgesics	Intravenous fluids (twice maintenance) Analgesics	Intravenous fluids (maintenance) Analgesics	Oral hydration Analgesics	Intravenous fluids (maintenance) Analgesics Vitamin D and calcium supplements
**Time from the onset of respiratory symptoms to the normalisation of the CPK level**	13 days	27 days	15 days	10 days	33 days

URTI: upper respiratory tract infection, CPK: creatine phosphokinase, MRC: Medical Research Council, SGPT - Serum Glutamic Pyruvic Transaminase, SGOT - Serum Glutamic-Oxaloacetic Transaminase

## Discussion

Severe leg pain and refusal to walk in a child can be distressing for both parents and physicians. The differential diagnosis for being unable to walk in a child is extensive, including neurologic, musculoskeletal, metabolic and psychiatric causes. Some of the serious conditions presenting with gait abnormalities include BACM, Guillain-Barre syndrome, transverse myelitis, rhabdomyolysis, dermatomyositis, poliomyelitis, deep venous thrombosis, muscular dystrophy and arthritis.^7,8^ Physicians may overlook BACM as a cause of refusal to walk due to its relatively lower incidence (2.6 cases per 100,000 children). The challenge in diagnosing BACM further increases with atypical presentations or courses. The exact pathogenesis of BACM remains unclear, although several theories exist. Muscle damage may arise from systemic, excessive and uncontrolled inflammation associated with a cytokine storm during viral infections, direct viral invasion of muscle tissue or immune-mediated damage.^9,10^ Muscle biopsy may demonstrate varying degrees of necrosis, ranging from scattered necrotic muscle fibres to widespread diffuse necrosis as seen in rhabdomyolysis. Depending on the timing of the biopsy, regenerating fibres may also be observed. In a majority of cases of viral myositis, no viral particles have been seen on biopsy.^11^

After the abovementioned serious conditions are ruled out, the diagnosis of BACM should be considered if the patient presents with calf pain and refusal to walk with a preceding viral infection. A majority of these cases are self-limiting, with complete recovery within 2 weeks. However, atypical presentations or courses can create diagnostic dilemmas, resulting in unnecessary investigations and delayed diagnosis and recovery. In our series, despite the atypical presentations, the final outcome was favourable due to early suspicion. Similar observations were reported by Brisca et al., Hyczko et al. and Kietaibl et al. in their studies.^1,4,5^ Brisca et al. found that 11% of children with acute myositis had toe walking. 1 The authors hypothesised that heel cord or muscle contracture secondary to viral infection and inflammation may be responsible for toe walking. It may also be a result of forced posture adopted by patients to decrease the pain in the gastrocnemius-soleus complex. Consistent with our findings, Brisca et al. reported that all children with toe walking returned to a normal gait upon follow-up.^1^ Recurrence of BACM, although reported in previous studies, is not common. Brisca et al. and Santos et al. found that 8.8% and 14.2% of cases had a recurrent attack of BACM, respectively.^1,12^ Recurrent episodes raise the possibility of genetic susceptibility in affected patients. Viral infections could act as a trigger and cause BACM in patients who are genetically susceptible with an underlying metabolic muscle defect. However, further studies are required to confirm this association. Notably, the second attack of BACM occurred due to a different viral infection. In their study, Ruff and Serist found that BACM usually recurred due to other viral infections.^13^ Further studies on a larger sample are required to determine whether this is a mere chance association. The delayed recovery seen in our case 5 could be due to concurrent severe vitamin D deficiency and hypocalcaemia. A similar case of BACM with delayed recovery was reported by Kentab and Kentab.^6^ Co-existing vitamin D deficiency and hypocalcaemia can cause proximal myopathy with weakness, resulting in delayed recovery as seen in our case. Thus, co-morbid conditions play an important role in recovery and should be addressed simultaneously. Although rhabdomyolysis may result from several causes, the classic triad of symptoms - weakness, brown-coloured urine and muscle pain - should raise suspicion for viral myositis. Viral myositis progressing to rhabdomyolysis is rarely seen in clinical practice. However, if a patient develops rhabdomyolysis due to BACM, monitoring for AKI and dyselectrolaemia is crucial. Most cases reported with BACM and rhabdomyolysis had good outcomes.^5^ Such patients should be managed with aggressive IVFs, urine alkalisation and possibly forced diuresis with loop diuretics. Renal replacement therapy should be considered in oligoanuric AKI.

## Conclusion

Physicians should maintain a heightened awareness of atypical presentations of BACM, particularly after ruling out more serious differential diagnoses. Consequently, primary care providers can avoid unnecessary investigations, promote timely diagnosis and offer appropriate counselling to parents. A practical approach includes thorough consideration of the full spectrum of symptoms, ensuring that even atypical signs are not overlooked. Additionally, routine screening for co-morbidities, such as vitamin D deficiency, should be incorporated, especially in cases with delayed recovery, to ensure comprehensive management at the primary care level.

## References

[1] Brisca G, Mariani M, Pirlo D (2021). Management and outcome of benign acute childhood myositis in pediatric emergency department.. Ital J Pediatr..

[2] Lundberg A (1957). Myalgia cruris epidemica.. Acta Paediatr..

[3] Crum-Cianflone NF (2008). Bacterial, fungal, parasitic and viral myositis.. Clin Microbiol Rev..

[4] Hyczko AV, Rohrbaugh MK, Suliman AK, Hackman NM (2021). A crawling case of benign acute childhood myositis.. SAGE Open Med Case Rep..

[5] Kietaibl AT, Fangmeyer-Binder M, Gondor G, Saemann M, Fasching P (2021). Acute viral myositis: profound rhabdomyolysis without acute kidney injury.. Wien Klin Wochenschr..

[6] Kentab AY, Kentab OY (2021). A unique presentation of benign acute childhood myositis in a child with influenza B.. Curr Pediatr Res..

[7] Terlizzi V, Improta F, Raia V (2014). Simple diagnosis of benign acute childhood myositis: lessons from a case report.. J Pediatr Neurosci..

[8] Ataullah AHM, De Jesus O (2022). StatPearls [Internet]..

[9] Costa Azevedo A, Costa E Silva A, Juliana Silva C (2022). Benign acute childhood myositis: a 5 year retrospective study.. Arch Pediatr..

[10] Cavagnaro SMF, Aird GA, Harwardt RI, Marambio QCG (2017). Miositis aguda benigna de la infancia: serie clfnica y revision de la literatura [Benign acute childhood myositis: clinical series and literature review].. Rev Chi Pediatr..

[11] Bhai S, Naddaf E, Dimachkie MM, Targoff IN, Shefner JM (2025). UptoDate..

[12] Santos JA, Albuquerque C, Lito D, Cunha F (2014). Benign acute childhood myositis: an alarming condition with an excellent prognosis!. Am J Emer Med..

[13] Ruff RL, Serist D (1982). Viral studies in benign acute childhood myositis.. Arch Neurol..

